# The Role of Hippocampal Interneuron Migration in Neurodevelopmental Disorders: A Systematic Review

**DOI:** 10.1002/hipo.70121

**Published:** 2026-08-17

**Authors:** M. A. C. Till, H. M. O. Reid, I. Murphy, B. R. Christie

**Affiliations:** ^1^ Department of Medical Neuroscience Dalhousie University Halifax Nova Scotia Canada; ^2^ School of Medical Sciences, University of Victoria Victoria British Columbia Canada; ^3^ Royal College of Surgeons in Ireland Dublin Ireland; ^4^ Island Medical Program, University of British Columbia Victoria British Columbia Canada; ^5^ Institute for Aging and Lifelong Health, University of Victoria Victoria British Columbia Canada; ^6^ Center for Behavioural Teratology, San Diego State University San Diego California USA

**Keywords:** dentate gyrus, GABA, hippocampus, interneuron, migration, neurodevelopment

## Abstract

Hippocampal abnormalities are frequently associated with neurodevelopmental disorders as interneurons are crucial in establishing the network connectivity of neurons. This systematic review analyzed primary literature with a focus on aberrant hippocampal interneuron migration as an etiology for neurodevelopmental disorders, summarizing the current field and identifying gaps that require further understanding. We adhered to the preferred reporting items for systematic reviews and meta‐analyses (PRISMA) guidelines to perform a comprehensive review of the existing research on GABAergic, parvalbumin, somatostatin, cholecystokinin, neuropeptide Y, calbindin, and calretinin interneuron migration in the hippocampus. Three blocks of search terms were used to extract 1459 papers from the PubMed, PsycINFO, and Web of Science databases. Following screening, we identified 29 articles which met the inclusion/exclusion criteria for the study. There is evidence that interneurons are affected in many neurodevelopmental conditions, with distinct regional and subtype‐specific differences. This systematic review has determined that there is a clear association between deficits in interneuron migration to the hippocampus and the presentation of neurodevelopmental disorders. The review highlights a significant lack of sex differences considered in the field, as well as a paucity of data for many specific neurodevelopmental conditions.

**Abbreviations**
ARND: alcohol‐related neurodevelopmental disorders
CA: corneau ammonis
CB: calbindin
CB1: cannabinoid type‐I receptors
CCK: cholecystokinin
CGE: caudal ganglionic eminence
CNS: central nervous system
CR: calretinin
DG: dentate gyrus
FASD: fetal alcohol spectrum disorder
GABA: gamma‐aminobutyric acid
H: hippocampus
HILs: hilar interneurons
HIPP cells: hilar‐perforant‐path‐associated interneurons
IN: interneuron
ISH: in situ hybridization
IvyCs: Ivy cells
IZ: intermediate zone
KI: knock‐in
KO: knock‐out
LTP: long‐term potentiation
MGE: medial ganglionic eminence
MZ: marginal zone
NGFCs: neurogliaform cells
NPY: neuropeptide Y
NS: not stated
OLM: oriens‐lacunosum moleculare
P: pallium
PRISMA: preferred reporting items for systematic reviews and meta‐analyses
PV: parvalbumin
qPCR: quantitative polymerase chain reaction
SOM: somatostatin
SP: subpallium
SZ: subventricular zone
THC: delta‐9‐tetrahydrocannabinol
TMP: tangential migration pathway
VT: ventral telencephalon

## 1 Introduction

Although neurodevelopmental disorders arise from diverse genetic and environmental causes, abnormalities in the hippocampus are common features of many conditions (Li et al. 2019). These include disorders with pronounced structural malformation, such as lissencephaly, as well as neuropsychiatric disorders, including schizophrenia, in which hippocampal dysfunction is well‐documented (Donmez et al. 2009; Heckers and Konradi 2010). Abnormal hippocampal interneuron migration has been linked to prenatal exposures, including maternal stress and cannabis use, and is associated with disorders like autism, Down syndrome, ATR‐X syndrome, and epilepsy (Berghuis et al. 2005; Colombo et al. 2007; Huo et al. 2018; Iida et al. 2024; Lawrence et al. 2010; Lussier and Stevens 2016). Given the hippocampus plays a critical role in learning and memory, understanding the mechanisms that regulate its development, including interneuron migration, is essential for identifying how developmental disruptions contribute to neurodevelopmental disorders.

Interneurons are the primary source of the neurotransmitter gamma‐aminobutyric acid (GABA) in the brain, which can modulate both excitatory (glutamatergic) and inhibitory (GABAergic) neurons (Pelkey et al. 2017). Interneurons make up 10%–15% of neurons in the hippocampus and have been shown to play a role in synaptic plasticity and learning and memory processes (Pelkey et al. 2017). They are found in a diversity of morphologies, with both local and long‐range projections, making them important for the integration of inputs from other brain regions (Booker and Vida 2018; Pelkey et al. 2017).

Interneurons are susceptible to developmental insults through multiple modalities, one of which is their migratory route during development. This route is called the tangential migratory pathway (TMP), and separates interneurons destined for the hippocampus from those migrating towards the cortex (Pelkey et al. 2017; Pleasure et al. 2000; Tricoire et al. 2011). The migratory pathway begins in either the medial ganglionic eminence (MGE) or the caudal ganglionic eminence (CGE) (Pelkey et al. 2017; Pleasure et al. 2000; Tricoire et al. 2011). Regardless of their initial location, interneurons will migrate through the marginal zone (MZ) and intermediate zone/subventricular zone (IZ/SZ) on their way to the hippocampal formation (Tricoire et al. 2011). While interneurons from the MGE and CGE both follow similar pathways, CGE‐derived interneurons are delayed compared to MGE‐derived interneurons (Miyoshi et al. 2010; Miyoshi et al. 2007; Tricoire et al. 2011). The CGE‐derived interneurons are 3 days behind the MGE‐derived interneurons, with interneuronogenesis peaking at E16.5 rather than E13.5, like their quicker counterparts (Miyoshi et al. 2010; Miyoshi et al. 2007). The first interneurons migrating from the MGE arrive at the developing hippocampus at E14.5 (Tricoire et al. 2011). The inhibitory interneurons will then distribute themselves throughout the hippocampal formation, with interneuron cell bodies present in multiple layers (Pelkey et al. 2017). This allows interneurons to integrate excitatory inputs from the hippocampal circuit and modulate their activity (Booker and Vida 2018; Pelkey et al. 2017). Once established in the hippocampus, interneurons face a sharp decline in their population between P0 to P30, following a period of critical plasticity where they help promote synaptogenesis (Pelkey et al. 2017; Tricoire et al. 2011).

Interneurons are a highly heterogeneous cell type, with separate subclasses assuming different roles in the hippocampus, and are characterized by varying morphologies and function (Booker and Vida 2018). Interneuron subtypes are typically observed using antibodies specific to the different markers which they express (Pelkey et al. 2017). It is crucial to consider differences in interneuron subtypes during neurodevelopment, since some are derived from the MGE, whereas others originate in the CGE. Parvalbumin (PV) interneurons, 60% of somatostatin (SOM) interneurons, Ivy cells (IvyCs), and some neurogliaform cells (NGFCs) all arise from the MGE (Pelkey et al. 2017; Pleasure et al. 2000; Tricoire et al. 2011). In contrast, 40% of SOM interneurons, cholecystokinin (CCK) interneurons, calretinin (CR) interneurons, and some NGFCs originate in the CGE (Pelkey et al. 2017; Pleasure et al. 2000; Tricoire et al. 2011). Experiments using Nkx2.1 mutant mice, which do not have MGE‐derived interneurons, exhibit an overall reduction of GABAergic‐INs in the hippocampus, suggesting that 60%–70% of hippocampal interneurons originate in the MGE (Pleasure et al. 2000).

Parvalbumin (PV) is a calcium binding protein which is expressed by some interneurons, establishing them as a separate subclass (Pelkey et al. 2017). PV is important during development and affects presynaptic calcium release (Collin et al. 2005). These interneurons typically synapse with the soma and proximal dendrites of pyramidal neurons, and occasionally other interneurons (Pelkey et al. 2017). PV‐INs are well established as being important for memory processes, as they are implicated in the spatial precision established by the granule cells of the dentate gyrus when it comes to novel environments, as well as the consolidation of memories following fear‐conditioning (Hainmueller et al. 2024; Ognjanovski et al. 2017). The role of PV‐INs in fear memory is likely related to their necessary activity for the generation of sharp wave ripples (Çaliskan et al. 2016; Hájos et al. 2013). PV‐INs are also crucial for other hippocampal rhythms, including the theta wave and theta‐gamma coupled oscillations (Wulff et al. 2009).

Somatostatin (SOM) is a neuropeptide that marks a heterogenous population of hippocampal interneurons. SOM is present in multiple cell types, including bistratified cells, cells located proximally to the alveus, as well as cells resembling typical oriens‐lacunosum moleculare (OLM) morphology with heterogenous targets (Chamberland et al. 2024). There are also multiple types of SOM‐INs in the dentate gyrus (DG), namely hilar‐perforant‐path‐associated interneurons (HIPP cells) and hilar interneurons (HILs), which govern different aspects of DG information flow (Yuan et al. 2017). Most SOM‐IN synapses are onto non‐GABAergic neurons, with few being onto other GABAergic‐INs of varying subtypes (Katona et al. 1999). SOM‐INs have also been implicated in the formation of memory ensembles and are involved in long‐term potentiation (LTP), as well as spatial navigation memories (Hainmueller et al. 2024; Racine et al. 2021; Royer et al. 2012; Stefanelli et al. 2016).

Cholecystokinin (CCK) expressing interneurons often have a basket cell morphology (Pelkey et al. 2017). These cells synapse onto the perisomatic and dendritic regions of excitatory cells (Pelkey et al. 2017). A defining feature of CCK‐INs is their high expression of cannabinoid type‐I receptors (CB1), implicating them in the endocannabinoid system found within the CNS (Pelkey et al. 2017). CCK‐INs also express other neuromodulators, like serotonin and acetylcholine, making them highly responsive to signaling and neural circuit activity (Cea‐del Rio et al. 2012). Consequently, CCK‐INs are important to different types of memory. They act to modulate the flow of information from the hippocampus to nucleus accumbens in context‐reward memories (Nguyen et al. 2024). CCK‐INs also regulate spatial memory and have been shown to tune place cells (Rangel Guerrero et al. 2024).

NPY is a neuropeptide which was originally identified as a gastric signaling molecule (Sundler et al. 1983). It is also located in the CNS and is used to visualize a few types of interneurons, specifically IvyCs and NGFCs (Pelkey et al. 2017). NGFCs are unique interneurons in that they interact with both the ionotropic GABA_A_ and metabotropic GABA_B_ receptors, resulting in slow and long‐lasting inhibitory signaling (Tamás et al. 2003). These cells are characterized by their high density of en passant boutons which release a cloud of non‐synapse specific GABA to nearby neurons (Bezaire and Soltesz 2013; Oláh et al. 2009; Pelkey et al. 2017). Their marker, NPY, acts on both presynaptic and postsynaptic receptors to mediate its effects (Sperk et al. 2007). While both receptor types are Gi_/o_ coupled, the Y1 receptor is predominantly postsynaptic, and the Y2 receptor is mainly presynaptic (Michel et al. 1998; Sperk et al. 2007). NPY‐expressing INs have been associated with adult neurogenesis, a process which occurs predominantly in the dentate gyrus (Decressac et al. 2009; Sperk et al. 2007). Interestingly, the NPY marker is commonly colocalized with SOM, suggesting that they label a similar population of interneurons (Freund and Buzsáki 1996). Notably, NPY has been established as a mediator for excitotoxicity as it is upregulated following the injection of kainate acid, used to induce seizures (Gruber et al. 1994; Smiałowska et al. 2003; Wu and Li 2005). This response is neuroprotective, likely due to its inhibitory actions (Smiałowska et al. 2003).

Calbindin (CB) and calretinin (CR) are calcium binding proteins that can be used to mark interneurons (Pelkey et al. 2017; Rogers 1987). These proteins are expressed in multiple brain regions and share a 58% sequence homology, which leads to cross‐reactivity concerns (Rogers 1987). However, it has been noted that CB and CR can label distinct interneurons, shown through variations in their morphology and location (Gulyás et al. 1996; Rogers 1987). Neurons marked by the colocalization of CB and CR are rare in the hippocampus and are mainly confined to the alveus and stratum oriens of the CA1 (Wouterlood et al. 2001). CB can label both GABAergic and glutamatergic neurons (Sloviter et al. 1991; Tóth and Freund 1992). Most granule cells of the dentate gyrus are CB+, as well as pyramidal cells of the CA1 and CA2 (Sloviter et al. 1991). CB+ cells are identified as INs mainly in the molecular layer, granule cell layer, and hilus, as well as subfields of the CA1 and CA2 (Sloviter et al. 1991). As a result, it is important to co‐label CB and GABA to ensure that the cells of interest are actually INs. The same issue arises for CR‐INs, as the marker also labels non‐pyramidal cells in all layers of the hippocampus, as well as in the granule cell layer and hilus (Jacobowitz and Winsky 1991; Résibois and Rogers 1992). INs positive for CR are found across all subfields of the CA (Jacobowitz and Winsky 1991). CR‐INs are notable in that they frequently synapse onto other neurons, providing a mechanism for disinhibition and further modulation of neural circuits (Pelkey et al. 2017). In this review, we consider the interneuron subtypes as they are immunohistochemically defined but acknowledge the potential for the colocalization of markers.

This review aims to synthesize the existing literature on hippocampal interneuron migration and neurodevelopmental disorders, while identifying any trends between subclasses of disorders. Previous work has highlighted the role of interneurons in the pathogenesis of neurodegenerative diseases, but not yet neurodevelopmental disorders (Reid et al. 2021). This review will guide future research on the topic by highlighting areas of consensus and areas which are under‐explored.

## 2 Methodology

The methodology for this review followed the PRISMA 2020 guidelines. However, the review protocol was not registered with PROSPERO. The search terms and inclusion/exclusion criteria were designed to select all primary, peer‐reviewed research papers describing how hippocampal interneuron migration is implicated in neurodevelopmental disorders (Table S1). The search was conducted using three search blocks separated by the “AND” operator, with individual terms separated by the “OR” operator (Table S2). The search was specific to the title/abstract, with no date restriction used. Using these search blocks, the M.T. searched three databases: PubMed, Web of Science, and PsycINFO. The citations were exported into the Covidence systematic review software (Veritas Health Innovation, Melbourne, Australia, 2025). The final search was conducted on November 10, 2025. Within Covidence, the duplicates were both automatically and manually removed, and all papers were screened according to the inclusion/exclusion criteria. First, the titles and abstracts of identified papers were screened independently by M.T. and I.M. Any conflicts were resolved independently by H.R. Full‐text reviews were then conducted independently by M.T. and H.R., with any conflicts being resolved through discussion. A forward and reverse search was completed from the included papers' citation list to identify other relevant papers not captured by the search terms. Any papers identified through the forward and reverse searches were immediately sent to full text screening. After the generation of the final list of included papers, relevant data was extracted from the selected papers by M.T., following a standard data collection template. Bias scoring was performed by M.T. following a modified version of the Scottish Intercollegiate Guidelines (Table S3) (“Scottish Intercollegiate Guidelines Network. Methodology Checklists” 2025). The studies were classified as either high quality, acceptable, or unacceptable.

## 3 Results

**FIGURE 1:**
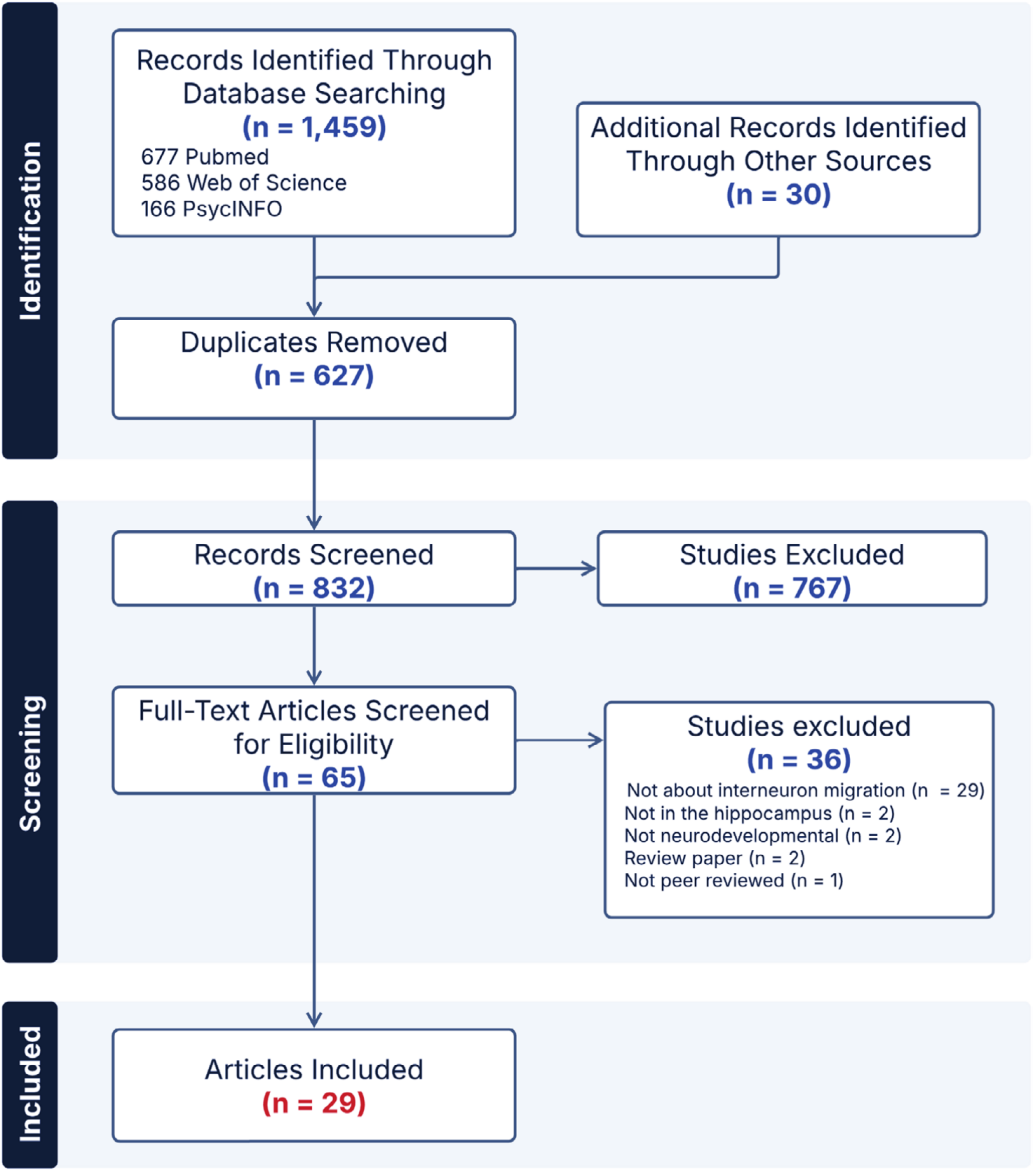
PRISMA flow diagram for the selection of articles in this systematic review.

Figure 1 displays a summary of the results. A total of 1459 studies were identified through database searching and additional sources. Following the removal of duplicates, the title, and abstract of 832 papers were screened. We identified 65 papers after the initial screening. Of these, 36 papers were excluded: 29 were not specific to interneuron migration, 2 were not specific to the hippocampus, 2 were review papers, 1 was not peer‐reviewed, and 2 were not related to any neurodevelopmental disorders. The excluded articles are referenced in Table S4. After the full text screening, 29 studies were included in the qualitative analysis. Of these 29 studies, 14 analyzed GABAergic‐INs, 21 analyzed PV‐INs, 9 analyzed SOM‐INs, CB‐INs, and CR‐INs, 4 analyzed NPY‐INs, and 2 analyzed CCK‐INs. Two studies which analyzed cells migrating from the MGE were also included.

Table 1 displays a summary of the disorders included in this review. Lissencephaly is the most studied neurodevelopmental disorder with five articles, followed by prenatal stress and schizophrenia with three articles each, autism with two articles, and one study published on each of the following: prenatal tetrahydrocannabinol (THC) exposure, prenatal valproic acid exposure, down syndrome, locoism, cognitive disabilities, developmental epileptic encephalopathy, hyperactivity coupled with cognitive impairment and spontaneous seizures, brominated flame retardant exposure, infantile spasms syndrome, ATR‐X syndrome, prenatal glycidol exposure, maternal separation, prenatal immune activation, irradiation‐induced microcephaly and/or epilepsy, delayed onset epilepsy, and prenatal gossypol exposure.

**TABLE 1:** Summary of disorders studied by included articles.

Categories	Disorders	Studies
Prenatal exposures	Prenatal stress exposure	Uchida et al. (2014)
		Lussier and Stevens (2016)
		Stevens et al. (2013)
	Prenatal THC exposure	Berghuis et al. (2005)
	Brominated flame retardant exposure	Saegusa et al. (2012)
	Prenatal glycidol exposure	Akane et al. (2013)
	Prenatal immune activation	Giovanoli et al. (2014)
	Prenatal gossypol exposure	Zhu et al. (2021)
	Prenatal swainsonine exposure	Liu et al. (2019)
	Prenatal valproic acid exposure	Shen et al. (2025)
Lissencephaly	Pafah1b1 haploinsufficiency	Dinday et al. (2017)
	Lis1 +/−	Fleck et al. (2000)
	Arx−/y; Pou3f4 and Dlx1/2	Fulp et al. (2008)
	Lis1 +/−	McManus et al. (2004)
	Arx mutant	Colombo et al. (2007)
Postnatal stress	Maternal separation	Kim et al. (2023)
Disorders affecting cognition	Down syndrome	Huo et al. (2018)
	Risk factor for cognitive disabilities	Eckert et al. (2021)
	Autism	Lawrence et al. (2010)
		Subramanian et al. (2024)
	ATR‐X syndrome	Seah et al. (2008)
Epilepsy	Developmental epileptic encephalopathy	Iida et al. (2024)
	Hyperactivity, cognitive impairment, and spontaneous epileptic seizures	Pennucci et al. (2016)
	Infantile spasms syndrome	Price et al. (2009)
	Irradiation induced microcephaly and/or epilepsy	Berden et al. (2025)
	Delayed onset epilepsy	Cobos et al. (2005)
Schizophrenia risk factors	Disc1 mutant	Lee et al. (2013)
	Disc1 mutant	Nakai et al. (2014)
	Disc1 mutant	Steinecke et al. (2012)

**FIGURE 2:**
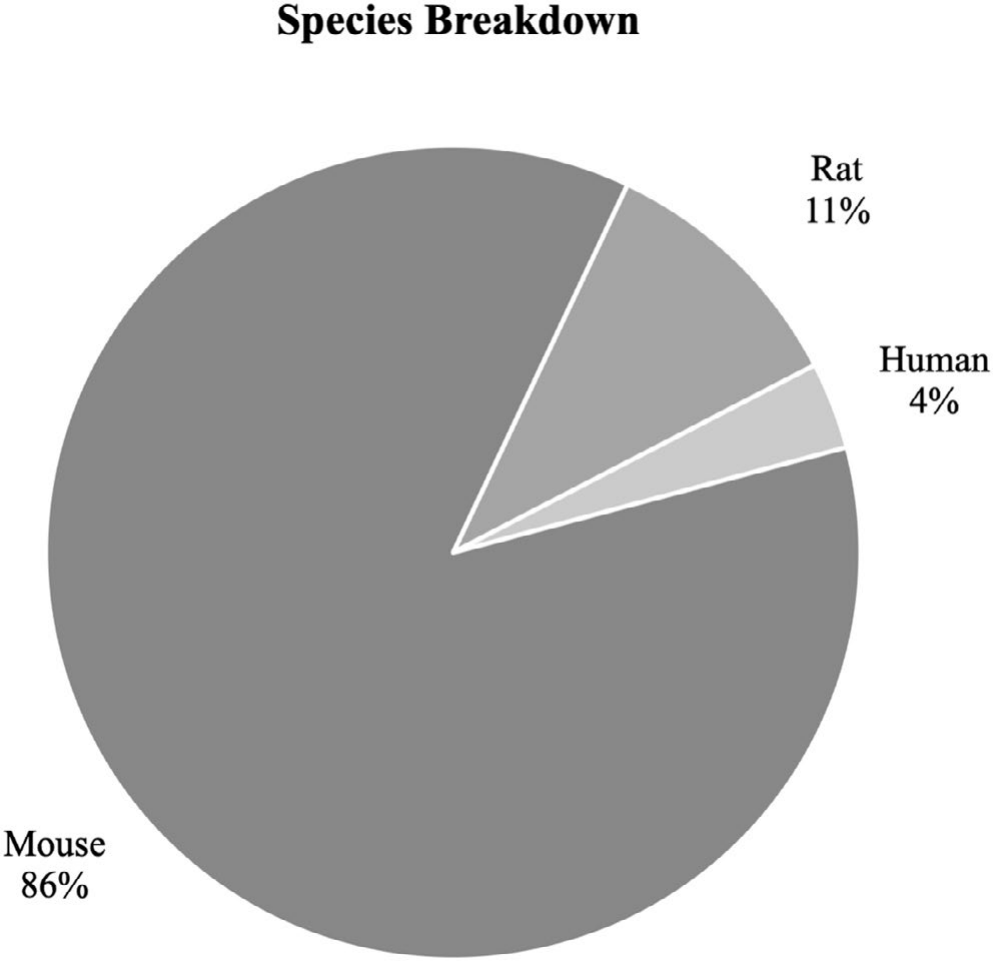
A breakdown of the species studied in the included papers.

**FIGURE 3:**
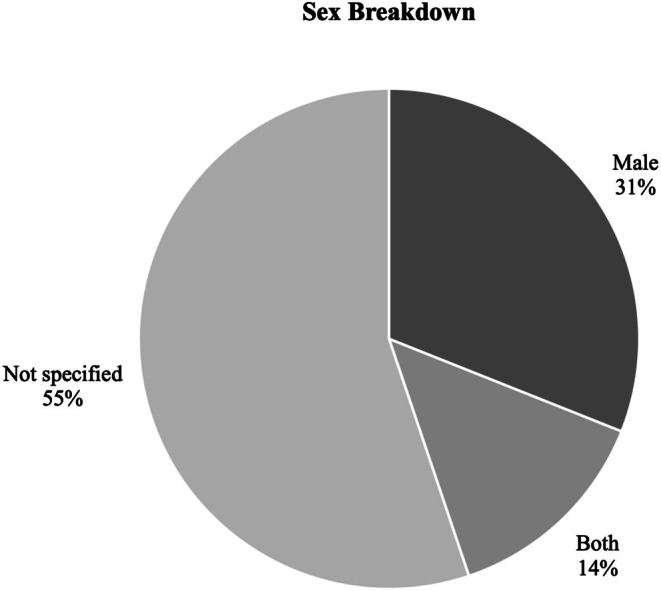
A breakdown of the sexes studied in the included papers.

Of these 29 articles, 25 used a mouse animal model, 3 used a rat animal model, and 1 used post‐mortem human tissue (Figure 2). Sex was rarely considered as a biological variable in the included studies. Among the included studies, 16 did not specify the sex of the animals used, 9 reported using only males, 4 reported using both sexes, and none using only females (Figure 3). Of the studies that included both males and females, one study reported finding no sex differences, two studies did not provide any data regarding sex differences, and one study found significant sex differences.

### 3.1 Bias Scoring

The results from the bias scoring illuminated some common trends amongst the included articles (Table S4). Only two of the 29 papers disclosed whether any animal deaths occurred because of the exposure model (Cobos et al. 2005; Iida et al. 2024). In addition, the bias scoring highlighted potential areas of inaccuracy in the articles, as just 28% of papers reported using a blinded experimenter in the assessment of the outcome. Positively, 41% of papers included the values of upper and lower limits in their statistical analyses. There is also a stark lack of sex differences considered in the results. Firstly, only 45% of studies reported the sex of the animals used. Of these studies, only two considered sex as a biological variable in their experimental design (Dinday et al. 2017; Nakai et al. 2014). One of the studies found significant sex differences between groups (Nakai et al. 2014). This result from the bias scoring highlights the need for both sexes to be included in studies on interneuron migration.

Three included studies were ranked as unacceptable according to the guidelines. Lussier and Stevens (2016) were deemed to be unacceptable due to a lack of transparent reporting. In this paper, no sample sizes or blinding were detailed. Similarly, Fleck et al. (2000) also did not report the sample sizes of their immunohistochemistry experiments. The paper also did not report the use of a blinded experimenter, nor did they discuss their methods for cell counting. In addition, the paper only shows histology figures without any graphical representation of their results. The results of this study should be considered as descriptive only. Finally, Steinecke et al. (2012) also did not report their methodology for cell counting and morphology experiments, resulting in an unacceptable ranking according to the bias scoring guidelines.

### 3.2 Prenatal Exposures

#### 3.2.1 Prenatal Stress Exposure

Three identified articles discussed prenatal stress exposure (Table 2). This exposure was studied as a developmental risk factor for neuropsychiatric disorders. Each of the three articles examined GABAergic interneurons using GAD67 as a marker, with two using GFP to visualize the positive cells. Two of the articles incorporated BrdU staining to examine the migration of newborn interneurons co‐labeled with GAD67 (Stevens et al. 2013; Uchida et al. 2014). One article found a decrease in BrdU+/GFP+ cells at E17.5 in the MGE, coupled with a reduction in Nkx2.1 expression, suggesting a reduction in newborn GABAergic‐INs (Uchida et al. 2014). In contrast, another article found no change in BrdU+/GFP+ cells at E13 in the ventral telencephalon following prenatal stress exposure (Stevens et al. 2013). However, the authors also observed a change in GAD67 immunoreactivity through the tangential migration pathway at E14. At P0, this difference was eliminated with no changes in GAD67/GFP+ cells in the DG or cornu ammonis (CA) (Stevens et al. 2013). However, one article observed no changes in GAD67+/GFP+ cells in the CA at any PND (Lussier and Stevens 2016). Although, when this result is coupled with changes in PV immunoreactivity, there was a significant reduction in the PV+/GAD67+ cell ratio in the CA at PD 24 and 150 (Lussier and Stevens 2016). Similarly, Uchida et al. (2014) observed a reduction in the PV+/GFP+ cell ratio in the CA1 at P21, with no changes in PV‐/GFP+ cells noted. However, the article also determined that in GAD67+/+ (wildtype) mice there was no loss of PV+ cells with maternal stress, suggesting that the heterozygous deletion of *Gad1*in the GAD67/GFP mice was important to the results (Uchida et al. 2014). This finding is similar to that of Lussier and Stevens (2016), which determined that there was no change in the number of PV+ cells in the CA at P24, but did note a reduction at P150. One article assessed changes in mRNA levels in the CA, finding no differences in the ratio of *pv*/*gad1* expression at P24, but a significant change at P150 (Lussier and Stevens 2016). Overall, these articles suggest that there are region and age specific changes in GABAergic and PV‐INs following prenatal stress exposure, yet no consensus can be reached.

**TABLE 2:** Included articles using prenatal exposure models.

	Species	Models	Sex	Assay types	Interneuron types	Regions	Findings
1	Mouse	Prenatal restraint and light stress	Male	Immuno	GABAergic	CA	No change in GAD67+ cells (Figure 1b/Table 1)
					PV		Decrease in PV+/GAD67+ ratio at P24 and P150 (Figure 2a/b)
				qPCR			No change in pv/gad1 expression at P24. Increase in pv/gad1 expression at P150 (Figure 2c/d)
2	Mouse	Prenatal restraint and light stress	NS	Immuno	GABAergic	MGE	Decrease in BrdU‐E15+/GFP+ cells (Figure 2f)
					PV	CA1	Decrease in the PV+/GFP+ ratio at P21 in maternally stressed GAD67+/GFP+ mice. No change in PV‐/GFP+ maternally stressed mice (Figure 5e/f)
						CA3	No change in PV+/GAD67+ ratio in GAD67+/GFP+ or PV‐/GFP+ maternally stressed mice (Figure 5e/f)
						DG	No change in PV+/GAD67+ ratio in GAD67+/GFP+ or PV‐/GFP+ maternally stressed mice (Figure 5e/f)
						H	No change in PV+/GAD67+ cells in GAD67+/+ maternally stressed mice (Figure S4C)
3	Mouse	Prenatal restraint and light stress	Male	Immuno	GABAergic	VT	No change in GAD67/GFP+ cells at E13 (Figure 1g). No change in BrdU‐E13‐GFP+ cells (Figure 5a)
						TMP	Altered distribution of GAD67/GFP+ cells at E14 (Figure 2a–d)
						DG	No change in GAD67/GFP+ cells at P0 (Figure 4c)
						CA	No change in GAD67/GFP+ cells at P0 (Figure 4c)
4	Rat	Prenatal THC exposure	NS	ISH	CCK	CA1‐CA3	Increase in CCK+ cell density at P2 in the strata radiatum and strata lacunosum‐moleculare (Figure 5a–c)
						Hilus	Increase in CCK+ cell density at P2 (Figure 5a–c)
5	Rat	Brominated flame retardant exposure	Male	Immuno	GABAergic	Hilus	No change in GAD67+ cells at P20 and P77 (Figure 3)
6	Rat	Prenatal glycidol exposure	Male	Immuno	PV	Hilus	No change in PV+ cells at P21 and P77 (Figure 5)
					CB		No change in Calb1+ cells at P21 and P77 (Figure 5)
					CR		Increase in Calb2+ cells at P21 and P77 (Figure 5)
7	Mouse	Prenatal immune activation	Male	Immuno	PV	CA1‐CA3	No change in PV+ cells at P70 (Figure 1)
						DG	No change in PV+ cells at P70 (Figure 1)
8	Mouse	Prenatal gossypol exposure	NS	Immuno	GABAergic	DG	No change in GAD67+ cells at P21 (Figure 4)
					PV		Increase in PV+ cells at P21 (Figure 4)
9	Mouse	Maternal swainsonine exposure	NS	Immuno	GABAergic	Hilus	No change in GAD67+ cells at P21 (Figure 5g/h)
					PV		No change in PV+ cells at P21 (Figure 5i/j)
10	Mouse	Prenatal valproic acid exposure	NS	Immuno	SOM	H	Decrease in SOM+ cells in animals exposed at E12.5 (Figure 5a/b)
						CA1	No change in SOM+ cells (Figure 5a/c)
						CA3	No change in SOM+ cells (Figure 5a/d)
						DG	Decrease in SOM+ cells in animals exposed at E10.5, E12.5, and E14.5 (Figure 5a/e)
					PV	H	No change in PV+ cells (Figure 5f/g)
						CA1	No change in PV+ cells (Figure 5f/h)
						CA3	No change in PV+ cells (Figure 5f/i)
						DG	No change in PV+ cells (Figure 5f/j)

*Note:* 1: Lussier and Stevens (2016); 2: Uchida et al. (2014); 3: Stevens et al. (2013); 4: Berghuis et al. (2005); 5: Saegusa et al. (2012); 6: Akane et al. (2013); 7: Giovanoli et al. (2014); 8: Zhu et al. (2021); 9: Liu et al. (2019); 10: Shen et al. (2025).

#### 3.2.2 Prenatal THC Exposure

Prenatal THC exposure was modeled in rats to study changes in CCK‐INs, which consisted of exposing dams to THC between GD5 and P2 (Table 2) (Berghuis et al. 2005). The authors noted a significant increase in CCK immunoreactivity in the strata radiatum and strata lacunosum‐moleculare of the CA and hilus at P2. This finding suggests that prenatal THC exposure induces an increase in CCK‐INs, which could be related to the high density of CB1 receptors localized to this interneuron subclass (Pelkey et al. 2017).

#### 3.2.3 Brominated Flame Retardant Exposure

One study observed the effects of brominated flame retardant (DBDE, TBBPA, and HBCD) exposure through the maternal diet from GD10 to P20 on GABAergic interneurons (Table 2) (Saegusa et al. 2012). Immunohistochemistry experiments assessing GAD67+ cells noted no changes in immunoreactivity for any flame retardant at both P20 and 77.

#### 3.2.4 Prenatal Glycidol Exposure

In one study, pregnant dams were administered glycidol through the drinking water between GD6 and P21 (Table 2) (Akane et al. 2013). The immunoreactivity of Calb1+, Calb2+ and PV+ cells was analyzed. The article found no changes in PV or Calb1 immunoreactivity at P21 or 77, suggesting that PV‐INs and CB+ cells are unaffected by glycidol exposure. However, the study noted an increase in Calb2+ cells in the hilus of rats exposed to the highest dose of glycidol, indicating that CR‐INs are affected.

#### 3.2.5 Prenatal Immune Activation

A single article observed the effects of prenatal immune activation through a poly(I:C) injection administered on GD9 on PV‐INs in mice (Table 2) (Giovanoli et al. 2014). The study observed no changes in PV immunoreactivity at P70 in the CA or DG of exposed animals.

#### 3.2.6 Prenatal Gossypol Exposure

One study orally exposed pregnant mice to gossypol daily beginning on E6.5 (Table 2) (Zhu et al. 2021). The authors observed an increase in PV+ cells and no changes in GAD67+ cells in the DG at P21. This finding suggests that gossypol exposure preferentially affects PV‐INs, yet other subclasses have yet to be observed.

#### 3.2.7 Prenatal Swainsonine Exposure

Swainsonine exposure can prompt the development of locoism (Liu et al. 2019). One article exposed pregnant mice to swainsonine from GD10 to P21 (Table 2) (Liu et al. 2019). Immunohistochemistry experiments were conducted, finding that swainsonine exposure did not alter the number of GAD67+ cells and PV+ cells. This indicates that interneuron migration may not be affected in this neurodevelopmental disorder.

#### 3.2.8 Prenatal Valproic Acid Exposure

Valproic acid is a frequently administered anti‐epileptic drug that is considered to be teratogenic (Tomson et al. 2016). One study evaluated the effects of prenatal valproic acid exposure on interneuron migration, finding that exposure at E12.5 decreased the expression of SOM‐INs in the hippocampus when evaluated at P42 (Table 2) (Shen et al. 2025). In addition, SOM‐IN levels were reduced in the dentate gyrus across exposure times (E10.5, E12.5, and E14.5). Prenatal valproic acid exposure did not alter PV‐IN counts in any hippocampal subregion. This finding raises the possibility that the effects are interneuron‐subtype specific.

### 3.3 Lissencephaly

Five articles examined changes in interneurons in an animal model of lissencephaly (Table 3). Two of the articles used mice with a heterozygous deletion of Lis1 (Fleck et al. 2000; McManus et al. 2004). One article using Lis1 mutant mice found a change in the distributions of PV+ cells and SOM+ cells in the CA1, and no changes in the distribution of CB+ cells and CR+ cells (Fleck et al. 2000). Interestingly, the other article using the Lis1 model noted a reduction in the migration distance and speed of CR+ cells (McManus et al. 2004). The authors also observed the same result in GABA+ cells (McManus et al. 2004). The differences in the results of these studies may be due to different ages examined, as Fleck et al. (2000) utilized adult mice, whereas McManus et al. (2004) obtained their data at E14.5.

**TABLE 3:** Included articles using Lissencephaly models.

	Species	Models	Sex	Assay types	Interneuron types	Regions	Findings
1	Mouse	Pafah1b1 haploinsufficiency	Both	Immuno	GABAergic	TMP	Delay in GAD67/GFP+ cell migration and disorganization (Figure 1a)
						DG	Decrease in GAD67/GFP+ cells at P5 and P30 in the DG (Figure 1B/C). Decrease in GAD67/GFP+ cells in the hilus and molecular layer at P30 (Figure 1D)
					PV		Decrease in PV+ cells at P30 (Figure 2D)
					SOM		Decrease in SOM+ cells at P30 (Figure 2E)
					CR		No change in CR+ cells at P30 (Figure 2F)
2	Mouse	Lis+/−	NS	Immuno	PV	CA1	Disruption in PV+ cells in the stratum pyramidale, yet they are anatomically similar (Figure 5A1/2)
					SOM		SOM+ cells are observed in stratum radiatum of the CA1 in mutants, not observed in WT (Figure 5D1/2)
					CR		No change in distribution (data not shown)
					CB		No change in distribution (data not shown)
3	Mouse	Lis+/−	NS	Immuno	GABAergic	TMP	Decrease in migration distance and speed at E14.5 (Figures 1C/F, 2B)
					CR		Decrease in migration distance and speed at E14.5 (Figures 1A/B/D/E, 2A)
4	Mouse	Arx−/y	NS	Microarray	CB	SP	Decrease in *calb1* at E14.5 (Figure 2B)
				ISH			Increase in *calb1* at E14.5 (Figure 3I)
		Dlx1/2 mutant					Increase in *calb1* at E14.5 (Figure 4B)
5	Mouse	Arx mutant	NS	In vitro migration assay	MGE‐derived cells	MGE	Decrease in mean migration distance. Changes in migrating cell morphology (Figure 9)

*Note:* 1: Dinday et al. (2017); 2: Fleck et al. (2000); 3: McManus et al. (2004); 4: Fulp et al. (2008); 5: Colombo et al. (2007).

Two articles used Arx mutant mice to model lissencephaly (Table 3) (Colombo et al. 2007; Fulp et al. 2008). One article analyzed *calb1* mRNA, finding reduced expression in the subpallium at E14.5 through microarray studies, but showed increased expression when detected through in situ hybridization (Fulp et al. 2008). The second article used MGE explants from mutant mice, finding a reduction in migration distance and changes in the morphology of MGE‐derived cells (Colombo et al. 2007). The contradictory results presented by Fulp et al. (2008) signal the need for further exploration on CB+ cells in lissencephaly models, however the altered behavior of MGE‐derived cells suggests that mutations in Arx affects the tangential migration of newborn interneurons (Colombo et al. 2007).

The article by Fulp et al. (2008) also used Dlx1/2 mutants to model lissencephaly, finding an increase in *Calb1* expression in the subpallium of mutants at E14.5. It should be noted that these results are descriptive and do not include quantitative data.

In addition, one article used a model of Pafah1b1 haploinsufficiency with Pafah1b1+/−; GAD67‐GFP mice (Table 3) (Dinday et al. 2017). The tangential migration of GABAergic‐INs was delayed and followed a disrupted pathway. These effects persisted to P5 and P30 in the DG, where a reduction in GAD67+ cell density was observed. This model also resulted in a decrease in PV and SOM immunoreactive cells at P30 in the DG, coupled with no changes in CR+ cells.

Overall, the variety of animal models used in the included studies on lissencephaly complicates the synthesis of the data collected, but the consensus amongst these studies is that the migration of interneurons of various subtypes is implicated in lissencephaly.

### 3.4 Postnatal Stress

One article studied the effects of maternal separation during the early developmental period of mice (Table 4) (Kim et al. 2023). The model involved separating the pups from the dam for 4 h each day, between P2‐20. The study observed an increase in GAD67+ cells in the CA1, CA3, DG, and hilus. Specifically, an increase in GAD67 immunoreactivity was noted in the molecular layer of the DG, the stratum oriens, stratum lacunosum‐moleculare, and stratum lucidum of the CA3, and the stratum oriens and stratum reticulata of the CA1. The study also labeled PV+ cells, also finding an overall increase in immunoreactivity in the CA1, CA3, DG, and hilus. There were some layer specific differences noted, with increased PV+ cells in the granule cell layer and molecular cell layer of the DG, the stratum oriens, stratum pyramidale, and stratum lacunosum‐moleculare of the CA3, as well as the stratum lacunosum‐moleculare of the CA1. These results demonstrate an overall increase in GABAergic‐INs that is specific to individual layers of the hippocampal formation, which likely also represents the change in PV‐IN distribution.

**TABLE 4:** Included articles using a postnatal stress model.

	Species	Model	Sex	Assay type	Region	Interneuron subtype	Finding
1	Mouse	Maternal separation	Male	Immuno	CA1	GABAergic	Overall increase in GAD67+ cells. Increase in specifically in the stratum oriens and stratum radiatum (Figure 3)
						PV	Overall increase in PV+ cells. Increase in specifically the stratum lacunosum‐moleculare (Figure 5)
					CA3	GABAergic	Overall increase in GAD67+ cells. Increase in specifically in the stratum oriens, stratum lacunosum‐moleculare, and stratum lucidum (Figure 3)
						PV	Overall increase in PV+ cells. Increase in specifically the stratum oriens, stratum pyramidale, and stratum lacunosum‐moleculare (Figure 5)
					DG	GABAergic	Overall increase in GAD67+ cells. Increase in specifically in the molecular layer and hilus (Figure 3)
						PV	Overall increase in PV+ cells. Increase in specifically the granule cell layer, molecular cell layer, and hilus (Figure 5)

*Note:* 1: Kim et al. (2023).

### 3.5 Neurodevelopmental Disorders Affecting Cognition

#### 3.5.1 Down Syndrome

One article evaluated the implications of interneuron migration in Down syndrome (Table 5) (Huo et al. 2018). This article used a mouse model where GABA progenitor clusters from human Down syndrome iPSC cell lines were injected into the medial septum. The animals were sacrificed 6 months following the transplant to perform immunohistochemistry experiments. Levels of Stem121+ cells, a marker for GABAergic human‐derived cells, were reduced. Stem121+ cells also migrated a shorter distance from the injection site to the hippocampus. Finally, the study noted reduced levels of Stem121+ SOM+ cells, CR+ cells, PV+ cells, and CB+ cells in the hippocampus of affected mice. However, amongst the Stem121+/GABA+ cells, there was an increase in DCX+ cells, indicating a decrease in interneuron maturity. This result was concluded from descriptive immunohistochemistry experiments. When considered together, these results suggest that the GABA progenitors isolated from humans with Down syndrome resulted in a deficiency in GABAergic interneuron migration, affecting many interneuron subtypes.

**TABLE 5:** Included articles using a model affecting cognition.

	Species	Models	Sex	Assay types	Interneuron types	Regions	Findings
1	Mouse	Down syndrome	NS	Immuno	GABAergic	H	Decrease in Stem121+/GABA+ cells in adults (Figure 4D/E)
							Reduction in migration distance from the medial septum to the hippocampus in adults (Figure 4F)
					PV		Very few + cells identified in adults (Figure S2C)
					SOM		Very few + cells identified in adults (Figure S2C)
					CR		Very few + cells identified in adults (Figure S2C)
					CB		Very few + cells identified in adults (Figure S2C)
2	Mouse	Cognitive disability	Both	Immuno	PV	H	No change in PV+ cells in adults (Figure 2B)
						CA1	No change in PV+ cells at P6‐10. Decrease at P14 (Figure 3A). Decrease in the stratum radiatum and stratum pyramidale in adults (Figure 2E)
						CA3	No change in PV+ cells at P6‐10. Decrease at P14 (Figure 3B) Decrease in the stratum radiatum and stratum pyramidale in adults (Figure 2H)
3	Human	Autism	Male	Immuno	CR	CA1	Increase in CR+ cells (Figure 5)
						CA2	No change in CR+ cells (Figure 5)
						CA3	No change in CR+ cells (Figure 5)
						DG	No change in CR+ cells (Figure 5)
						Anterior H	No change in CR+ cells (Figure 5)
					PV	CA3	Increase in PV+ cells (Figure 3)
						Anterior H	Increase in PV+ cells (Figure 3)
						CA1	Increase in PV+ cells (Figure 3)
						DG	No change in PV+ cells (Figure 3)
						CA2	No change in PV+ cells (Figure 3)
					CB	DG	Increase in CB+ cells (Figure 4)
						CA3	No change in CB+ cells (Figure 4)
						CA2	No change in CB+ cells (Figure 4)
						CA1	No change in CB+ cells (Figure 4)
						Anterior H	No change in CB+ cells (Figure 4)
4	Mouse	Autism	Both	Immuno	PV	CA1	Decrease in PV+ cells in adults (Figure 1I)
						CA2	No change in PV+ cells in adults (Figure 1I)
						CA3	No change in PV+ cells in adults (Figure 1I)
						DG	No change in PV+ cells in adults (Figure 1I)
						H	Overall decrease in PV+ cells in adults (Figure 1H)
					SOM	CA1	Decrease in SOM+ cells in adults (Figure 2F)
						CA2	No change in SOM+ cells in adults (Figure 2F)
						CA3	No change in SOM+ cells in adults (Figure 2F)
						DG	No change in SOM+ cells in adults (Figure 2F)
						H	Overall decrease in SOM+ cells in adults (Figure 2E)
					NPY	CA1	Decrease in NPY+ cells in adults (Figure 2L)
						CA2	No change in NPY+ cells in adults (Figure 2L)
						CA3	No change in NPY+ cells in adults (Figure 2L)
						DG	No change in NPY+ cells in adults (Figure 2L)
						H	Overall decrease in NPY+ cells in adults (Figure 2K)
5	Mouse	ATR‐X syndrome	NS	qPCR	NPY	Forebrain	Reduction in NPY transcripts at E13.5 and P0.5 (Figure 5A)
					SOM		Reduction in SOM transcripts at E13.5 and P0.5 (Figure 5A)
					CCK		Reduction in CCK transcripts at P0.5 (Figure 5A)
				Immuno	NPY	VT	Reduction in NPY+ cells at E13.5 and E16.5 (Figure 5B)

*Note:* 1: Huo et al. (2018); 2: Eckert et al. (2021); 3: Lawrence et al. (2010); 4: Subramanian et al. (2024); 5: Seah et al. (2008).

#### 3.5.2 Risk Factor for Cognitive Disabilities

A study used BDNF‐Pax2 KO mice to develop a model of cognitive disability (Table 5) (Eckert et al. 2021). This article examined PV immunoreactivity, noting no changes in PV+ cells in the whole hippocampus of adult mice. However, when the hippocampal subfields were examined, the article found a reduction in PV+ cells in the stratum pyramidale and stratum radiatum of the CA1 and CA3 of adults. At animals aged P6‐10 there was no change in PV+ cells in either the CA1 or CA3. At P14, a reduction in PV+ cells in the CA1 and CA3 were noted, suggesting that deficits in interneuron migration in this animal model are specific to the hippocampal subfield and age of the animals studied.

#### 3.5.3 Autism

Two articles assessed the relationship between interneuron migration and autism (Table 5). One article used post‐mortem human tissue and the other used a Nrp2‐KO mouse model (Lawrence et al. 2010; Subramanian et al. 2024). Both studies evaluated PV immunoreactivity, with the human model article finding an increase in PV+ cells in the CA1 and CA3, and the mouse model article finding a reduction in PV+ cells in the CA1, with no changes in the DG or CA3. With the Nrp2‐KO model, a reduction in SOM+ cells was observed in the CA1, coupled with a reduction in NPY+ cells in the same subfield (Subramanian et al. 2024). In the human tissue, an increase in CB+ cells was observed in the DG, as well as an increase in CR+ cells in the CA1 (Lawrence et al. 2010). The opposing trends in interneuron marker expression is likely attributable to the different species used in the studies.

#### 3.5.4 ATR‐X Syndrome

One article modeled ATR‐X syndrome using Atrx‐Foxg1‐Cre mice (Table 5) (Seah et al. 2008). This study used PCR to identify a reduction in NPY transcripts in the forebrain at E13.5 and P0.5, a reduction in SOM transcripts in the forebrain at E13.5 and P0.5, as well as a reduction in CCK transcripts in the forebrain at P0.5. The NPY interneuron result was verified by immunohistochemistry, finding a reduction in NPY+ cells in the ventral telencephalon of both E13.5 and E16.5 animals.

### 3.6 Epilepsy

#### 3.6.1 Developmental Epileptic Encephalopathy

A girdin‐KO mouse model was used in one article to represent developmental epileptic encephalopathy (Table 6) (Iida et al. 2024). Immunohistochemistry results found a decrease in the density of GAD65/67+ cells in the whole hippocampus at P7. When considering individual subtypes, the study determined that there was a decrease in PV+ cells and SOM+ cells in the DG and CA of P28 animals.

**TABLE 6:** Included articles using a model of epilepsy.

	Species	Models	Sex	Assay types	Interneuron types	Regions	Findings
1	Mouse	Developmental epileptic encephalopathy	NS	Immuno	GABAergic	H	Decrease in GAD65/67+ cells at P7 (Figure 5C,D)
					PV	CA	Decrease in PV+ cells at P28 (Figure 5E–G)
						DG	Decrease in PV+ cells at P28 (Figure 5E–G)
					SOM	CA	Decrease in SOM+ cells at P28 (Figure S15)
						DG	Decrease in SOM+ cells at P28 (Figure S15)
2	Mouse	Hyperactivity, cognitive impairment, and spontaneous epileptic seizures	NS	Immuno	PV	H	Decrease in PV+ cells in both Rac3KO and Rac1N adults (Figure 4B,C).
						CA1	Decrease in PV+ cells in Rac1N and Rac3KO adults (Figure 4B,C). Decrease in colocalized PV+/GAD67+ cells in the stratum pyramidale of Rac1N and Rac3KO adults (Figure 5C)
						CA3	No change in PV+ cells in Rac1N adults. Decrease in PV+ cells in Rac3KO adults (Figure 4B,C)
						DG	No change in PV+ cells in Rac1N adults. Decrease in PV+ cells in Rac3KO adults (Figure 4B,C)
3	Mouse	Infantile spasms syndrome	NS	Immuno	PV	H	No change in PV+ cells in adults (data not shown)
					CR		No change in CR+ cells in adults (data not shown)
					NPY		No change in NPY+ cells in adults (Table 1)
					CB		No change in CB+ cells in adults (Figure 6E,F, Table 1)
						DG	Decrease in CB+ cells in the granule cell layer of adults (Figure 6E,F)
4	Mouse	Irradiation induced microcephaly and/or epilepsy	NS	RNAseq	MGE‐derived cells	MGE	Downregulation in migration‐related processes (Figure 4C)
				Live‐cell imaging		TMP	Brain slices at E13.5 show slower interneuron migration in the MZ and IZ (Figure 5L,N). MGE explants show a decrease in the average migration speed (Figure 6B)
				Immuno			Decrease in GFP+/BrdU+ cells at the P‐SP boundary in the MZ, SP, IZ/SVZ at E15 (Figure 5G). Decrease in GFP+/BrdU‐ cells in the SP at the P‐SP boundary (Figure 5H). No changes at E13 (Figure 5C,D)
					PV	H	No change in PV+ cells at P56 (Figure 8Q)
					SOM	H	No change in PV+ cells at P56 (Figure 8S)
5	Mouse	Delayed onset epilepsy	NS	Immuno	GABAergic	CA1	No change in GAD67+ cells at P12 (data not shown). Decrease at 2 months (Figure 2M). Decrease remained at 6 months (Figure 2M)
						CA3	No change in GAD67+ cells at P12 (data not shown). Decrease at 2 months (in text)
						DG	No change in GAD67+ cells at P12 (data not shown). Decrease at 2 months (in text)
					SOM	CA1	No change in SOM+ cells at P12. Decrease in SOM+ cells at 1–2 months (Figure 3S)
					CR	CA1	No change in CR+ cells at P12. Decrease in CR+ cells at 1–2 months (Figure 3S)
					NPY		No change in NPY+ cells at P12. Decrease in NPY+ cells at 1–2 months (Figure 3S)
					CB		No change in CB+ cells (Figure S2K)
					PV		No change in PV+ cells (Figure S2K)

*Note:* 1: Iida et al. (2024); 2: Pennucci et al. (2016); 3: Price et al. (2009); 4: Berden et al. (2025); 5: Cobos et al. (2005).

#### 3.6.2 Hyperactivity, Cognitive Impairment, and Spontaneous Epileptic Seizures

One article used Rac3‐KO and Rac1‐conditional KO (Rac1N) mouse models to create a disease phenotype showing altered behavior such as hyperactivity, cognitive impairment, and spontaneous epileptic seizures (Table 6) (Pennucci et al. 2016). While this phenotype does not directly resemble a single human neurodevelopmental disorder, its symptoms are a result of the KO model, and hence are considered in this review. The study found region and mutant specific changes in PV‐INs. The results showed a decrease in overall PV+ cells in the hippocampus in both mutants. Rac1N mice presented with a decrease in PV+ cells in the CA1, with the DG and CA3 remaining unaffected. On the other hand, the Rac3KO mice presented with a reduction in both CA1 and DG PV+ cells. Similar to the Rac1N mice, the Rac3KO mice showed no change in PV immunoreactivity in the CA3. GABAergic‐INs were also measured, finding a decrease in PV+/GAD67+ cells in the stratum pyramidale of the CA1 of both mutants. These findings suggest that the overall trend is towards a reduction in PV‐INs, however the regional distribution of these cells may be affected by the mouse model.

#### 3.6.3 Infantile Spasms Syndrome

Arx(GCG)_10_+7 polyalanine expansion KI mutant mice were used in one study to model infantile spasms syndrome (Table 6) (Price et al. 2009). PV, CR, and CB interneurons were shown to be unaffected by the mutation. However, CB+ cells were reduced in the granule cell layer of the adult DG. This result points to a region and subtype specific effect.

#### 3.6.4 Irradiation Induced Microcephaly and/or Epilepsy

One article examined how interneuron migration is implicated in irradiation‐induced microcephaly and/or epilepsy using mice exposed to a single dose of radiation at E11 (Table 6) (Berden et al. 2025). MGE‐derived cells were visualized using Nkx2.1‐GFP mice. RNAseq showed a downregulation in migration‐related processes in MGE‐derived cells. A BrdU injection 30 min prior to the radiation exposure allowed for the visualization of newborn cells. The study details a reduction in GFP+/BrdU+ cells at the pallium–subpallium boundary in the subpallium, MZ, and IZ/SVZ at E15. There was also a decrease in GFP+/BrdU− cells at the pallium–subpallium boundary in the subpallium, with no changes noted in the MZ or IZ/SVZ. The same studies were also performed at E13 with no changes noted. In addition, live‐cell imaging obtained from E13.5 brain slices found a reduction in interneuron migration in speed in both the active and resting phases, specific to the MZ and IZ. Data obtained from MGE explants found a similar result, showing a reduction in the migration speed as well as instantaneous speed of cells migrating out of the explant. The study also examined individual interneuron subtypes, finding no changes in both hippocampal PV+ cells and SOM+ cells at P56. This result may reflect that the migration of the interneurons is impaired, but not their maturation to express their respective markers.

#### 3.6.5 Delayed Onset Epilepsy

A single study used Dlx1−/− mutant mice to study delayed onset epilepsy (Table 6) (Cobos et al. 2005). Immunohistochemistry found an overall decrease in GAD67+ cells in the DG, CA1, and CA3 of 2‐month‐old mutants. The study then examined individual subtypes, noting no changes in SOM+ cells, CR+ cells, and NPY+ cells at P12. At 2‐months, the mutants expressed a reduction in SOM+ cells, CR+ cells, and NPY+ cells in the CA1. PV+ cells and CB+ cells were not affected at any timepoint. This finding likely represents a deficit in interneuron maturation and distribution rather than migration, due to the differences between groups only appearing towards adolescence.

### 3.7 Schizophrenia Risk Factors

Three articles used Disc1 mutant mice to model interneuron migration changes in schizophrenia (Table 7) (Lee et al. 2013; Nakai et al. 2014; Steinecke et al. 2012). Two studies examined PV‐INs, with Lee et al. (2013) showing an increase in PV+ cells in the CA1 and CA2/3, and Nakai et al. (2014) finding a decrease in PV+ cells in females, with no changes noted in males. These sex differences were eliminated when the results were compared within the CA1, CA3, and DG, rather than the whole hippocampal formation. In addition, one article found a decrease in GAD67+/PV+ cell colocalization (Lee et al. 2013). These authors also found no changes in CB+ cells in the hippocampus, but did observe alterations in CB+ cell distribution along the TMP. Hence, the migration of CB+ cells may be affected in Disc1 mice, but any differences appear to resolve when the cells reach the hippocampus. One article examined MGE‐derived cells using MGE explants from Disc1 mice, finding an overall reduction in migration from mutant explants (Steinecke et al. 2012).

**TABLE 7:** Included articles using a model of Schizophrenia.

	Species	Models	Sex	Assay types	Interneuron types	Regions	Findings
1	Mouse	Disc1 mutant	NS	Immuno	PV	CA1	Increase in PV+ cells in adults (Figure 6B)
						CA2/3	Increase in PV+ cells in adults (Figure 6B)
						DG	No change in PV+ cells in adults (Figure 6B)
					GABAergic	H	Decrease in GAD67+/PV+ cells in adults (Figure 5B)
					CB	CA1	No changes in CB+ cells in adults (Figure 6C)
						CA2/3	No changes in CB+ cells in adults (Figure 6C)
						TMP	Altered distribution of CB+ cells during migration at E14 and E16 (Figure 1)
2	Mouse	Disc1 mutant	Both	Immuno	PV	H	Decrease in PV+ cells in females. No changes in males. No differences within subfields (Figure 1B/C)
3	Mouse	Disc1 mutant	NS	Cell migration assay	MGE‐derived cells	MGE	Decrease in cells migrating from MGE explants (Figure 3)

*Note:* 1: Lee et al. (2013); 2: Nakai et al. (2014); 3: Steinecke et al. (2012).

## 4 Discussion

### 4.1 Prenatal Exposures

Three articles used a model of prenatal stress exposure. GABAergic‐INs were most studied, and preliminary results suggest that there could be a time delay following the stress exposure before deficits in GABAergic‐INs are seen, but the decrease may be rescued at later time points (P24‐P150) (Lussier and Stevens 2016; Stevens et al. 2013; Uchida et al. 2014). The results on PV‐INs are limited to those concluded by one study but may indicate a longer‐term deficit (Lussier and Stevens 2016). The discrepancies between GABAergic and PV‐INs indicate that there are likely region‐specific effects or alterations in interneuron maturation. Unfortunately, other interneuron subtypes have not yet been evaluated in a prenatal stress model. It would be valuable for future works to correlate interneuron subtype changes with behavioral testing to determine the phenotype resulting from interneuron migration disruption.

One article studied the effects of prenatal THC exposure on interneuron migration, noting an increase in CCK‐INs (Berghuis et al. 2005). This finding is consistent given the previously established role of CCK‐INs in the endocannabinoid system due to their high expression of the CB1 receptor (Pelkey et al. 2017). As THC crosses the placental barrier and acts as an agonist at the CB1 receptor, it could decrease GABA release from CCK‐INs (Hutchings et al. 1989; Laaris et al. 2010). This has special implications as GABA is excitatory during embryonic development and the first postnatal week in rodents (Valeeva et al. 2013; Wu and Sun 2015). The consequent reduction in excitatory GABAergic signaling may alter activity‐dependent hippocampal circuitry and prompt the reorganization and differential migration of CCK‐INs. Indeed, previous work has identified that acute CB1 receptor overactivation during development suppresses hippocampal network activity (Bernard et al. 2005). Migration differences in other interneuron subtypes should be evaluated to determine the extent of prenatal THC exposure's impact on interneuron development.

Prenatal valproic acid exposure can lead to a diagnosis of fetal valproate spectrum disorder in some individuals (Bluett‐Duncan et al. 2023). The disorder is characterized by cognitive impairments and an increase in the risk of autism spectrum disorder and attention‐deficit hyperactive disorder (Bluett‐Duncan et al. 2023). Prenatal valproic acid exposure was found to decrease the expression of SOM‐INs in the hippocampus and dentate gyrus while leaving PV‐INs unaffected (Shen et al. 2025). The teratogenic effects of valproic acid may be restricted to certain interneuron subtypes, and future studies should examine others to determine the drug's overall effect. These findings should be correlated with behavioral changes to determine whether changes in interneuron migration may contribute to the neurodevelopmental phenotype seen in fetal valproate spectrum disorder.

Articles describing prenatal exposure to brominated flame retardants, glycidol, immune activation, gossypol, and swainsonine found no changes in interneuron migration (Akane et al. 2013; Giovanoli et al. 2014; Liu et al. 2019; Saegusa et al. 2012; Zhu et al. 2021). This result suggests that there may be additional mechanisms underlying the abnormalities associated with these conditions that warrant further investigation.

### 4.2 Lissencephaly

Lissencephaly is characterized by malformation of the cerebral gyri and structural abnormalities of the cortex, and so it follows that cell migration is greatly affected (Koenig et al. 2021). It appears to disrupt interneuron migration and differentiation despite differences in the genetic models used to study the condition. Amongst the five articles included in the review, four different mouse models were used, and similar results were noted across the studies. This finding suggests the presence of a common developmental mechanism in lissencephaly. There appears to be a reduction in GABAergic‐IN migration, coupled with decreased GABAergic‐IN levels postnatally (Colombo et al. 2007; Dinday et al. 2017; McManus et al. 2004). As well, PV‐INs and SOM‐INs are affected in this condition (Dinday et al. 2017; Fleck et al. 2000). One article found a decrease in the migration of CR‐INs (McManus et al. 2004), with a second article observing no changes in CR immunoreactivity at P30 (Dinday et al. 2017). The discrepancy of this finding is likely a result of the different timepoints studied, and indicates a potential developmental timing effect. Finally, there are mixed results on the expression of *calb1*, with one article noting both increased and decreased *calb1* depending on the experimental technique (Fulp et al. 2008). Ultimately, it appears that lissencephaly affects interneurons across multiple subtypes, and future work is required to clarify the disruption to CB‐ and CR‐INs. Interneuron abnormalities arising in early development may provide a mechanistic basis for the network dysfunction and epileptogenesis commonly associated with lissencephaly, and future work should investigate whether this could be a causal relationship (Katsarou et al. 2017).

### 4.3 Postnatal Stress

The finding that postnatal stress increased both GABAergic‐ and PV‐INs demonstrates the susceptibility of inhibitory circuits to environmental influences (Kim et al. 2023). These results suggest that adverse early‐life experiences can alter interneuron development by increasing the abundance of specific subtypes. This is in stark contrast to the reviewed studies on prenatal stress, which found a general decrease in interneuron populations (Lussier and Stevens 2016; Stevens et al. 2013; Uchida et al. 2014). Overall, this indicates a differential effect of early life stress depending on the stage at which it occurred. Prenatal stress may preferentially affect interneuron migration and differentiation, whereas postnatal stress may induce changes in inhibitory circuitry at a later developmental timepoint.

### 4.4 Neurodevelopmental Disorders Affecting Cognition

Interneuron migration changes have been noted across multiple neurodevelopmental disorders affecting cognition; however the directionality and characteristics of these changes vary by condition and model system.

A general reduction in hippocampal interneurons has been noted in both Down syndrome and ATR‐X syndrome. More general decreases in GABAergic‐INs were found in Down syndrome, whereas subtype‐specific changes in NPY‐, CCK‐, and SOM‐INs were identified in ATR‐X syndrome (Huo et al. 2018; Seah et al. 2008).

Some neurodevelopmental disorders identified region and developmental timing effects. In a model of risk factors for cognitive disability, a reduction in PV‐INs was identified specifically in the CA1 and DG, but not when the whole hippocampal formation was analyzed (Eckert et al. 2021). Additionally, the study only found these deficits at P14 and in adults, but not at earlier timepoints. This suggests that the model's effects on interneurons are delayed and more so related to organization rather than migration.

In models of autism spectrum disorder there is a lack of consensus as to the directionality of interneuron subtype changes. Lawrence et al. (2010) identified an increase in CR‐INs, PV‐INs, and CB+ cells in post‐mortem human brain tissue. However, in a mouse model, Subramanian et al. (2024) found a decrease in PV‐INs, SOM‐INs, and NPY‐INs. While the studies clearly identify that interneurons are disrupted in autism, it remains unclear how they are affected and what the underlying mechanism is. The opposing results may be a consequence of the different species studied, or could indicate a heterogeneity in the condition.

Overall, these studies provide evidence that abnormalities in interneuron migration are a recurring feature across neurodevelopmental disorders affecting cognition. Although the nature of these changes depends on the interneuron subtype, brain region, developmental timepoint, as well as the model used.

### 4.5 Epilepsy

Interneurons are disrupted across diverse models of epilepsy and epilepsy‐associated neurodevelopmental disorders. In general, a reduction in hippocampal interneurons was a consistent finding across studies. No studies found an increase in any interneuron subtype.

GABAergic‐INs were found to be decreased in a model of developmental epileptic encephalopathy and hyperactivity, cognitive impairment, and spontaneous epileptic seizures, as well as PV‐ and SOM‐INs in some cases (Iida et al. 2024; Pennucci et al. 2016). A similar study modeling irradiation‐induced microcephaly and/or epilepsy also identified a reduction in GABAergic‐INs but no changes in PV or SOM subtypes (Berden et al. 2025). This suggests that there may be a uniform decrease in inhibition provided by hippocampal interneurons, but there is a lack of commonality amongst subtypes.

There may also be developmental timepoint‐specific alterations in interneurons. Cobos et al. (2005) found that GABAergic, SOM, and CR‐INs were unaffected at P12 in a model of delayed onset epilepsy, but then deficits appeared at 2 months. Interestingly, the decrease in GABAergic‐INs was reversed by 6 months. These findings indicate that interneurons remain plastic through early development.

Other interneuron subtypes have also been evaluated in epilepsy models. No changes in NPY‐INs were identified. The effects on CB+ cells may be region‐dependent, as Price et al. (2009) revealed a reduction in the granule cell layer of the adult DG, but not when the whole hippocampal formation was analyzed in combination.

When taken together, these findings suggest a general deficit in GABAergic‐INs in conditions characterized by epilepsy; however no consensus can be made as to the involvement of specific subtypes.

### 4.6 Schizophrenia

Studies modeling schizophrenia with disc1 mutation models have identified interneuron subtype deficits and migration differences. PV‐INs appear to be specifically vulnerable; however no studies have examined GABAergic‐INs in general, and the investigation of other subtypes is limited. Both an increase and decrease in PV‐INs has been noted in the disc1 model (Lee et al. 2013; Nakai et al. 2014). The decrease was specific to females, whereas no information on sex was provided in the study that found an increase in PV‐INs. There may be a sex‐dependent effect of schizophrenia on PV‐INs which needs to be elucidated. The effects on CB+ cells also remain unclear as no changes were found in the hippocampal formation of adults, but migration deficits were seen along the TMP in embryonic development (Lee et al. 2013). A similar result was replicated using MGE explants, where a decrease in cells migrating outwards was found (Steinecke et al. 2012). These results indicate that the migration of some interneuron subtypes are preferentially affected in disc1 mutants.

### 4.7 Future Directions

Certain neurodevelopmental disorders are notably missing from the literature. For example, fetal alcohol spectrum disorder (FASD) and alcohol‐related neurodevelopmental disorders (ARND) have been frequently associated with hippocampal abnormalities (Astley et al. 2009; Coles et al. 2011; Dudek et al. 2014; Willoughby et al. 2008), yet to our knowledge no research has been conducted on the contribution of disrupted interneuron migration to these pathologies.

The field of interneuron migration has become important not only for understanding the underlying pathology of many neurodevelopmental disorders, but also in developing novel treatments. While out of the scope of this review, interneuron‐targeting treatments require a baseline understanding of which deficits exist for which condition. One neuropsychiatric disorder that has shown potential for interneuron‐targeting treatments is schizophrenia. Postmortem human studies have found a deficit in PV‐INs in the hippocampus of individuals affected by schizophrenia (Zhang and Reynolds 2002). Interestingly, new treatments aim to target this deficit by implanting MGE‐derived interneuron transplants into the hippocampus (Donegan et al. 2017; Perez and Lodge 2013). These transplanted interneurons incorporate with endogenous hippocampal circuitry and can regulate hippocampal activity, dopamine neuron activity, as well as alleviate some behavioral effects of schizophrenia (Donegan et al. 2017; Perez and Lodge 2013). To further the translational research conducted in the field of interneuron migration, future studies should consider this methodology and determine if it could be a feasible therapeutic option to treat other neurodevelopmental disorders.

This PRISMA review establishes a clear correlation between hippocampal interneuron migration and the pathology of neurodevelopmental disorders. Further research should consider sex as a biological variable, as it remains an important yet often overlooked factor in current literature. This review also highlights many neurodevelopmental disorders and associated risk factors that have received limited investigation, offering many avenues for future studies to further clarify their effects on interneuron migration.

## Author Contributions

**M.A.C. Till:** conceptualization, methodology, investigation, writing – original draft, writing – review and editing, visualization, project administration. **H.M.O. Reid:** conceptualization, methodology, investigation, writing – review and editing, project administration. **I. Murphy:** investigation, writing – review and editing. **B.R. Christie:** conceptualization, writing – review and editing, project administration, supervision, funding acquisition.

## Funding

This work was supported by the Canadian Institutes of Health Research (CIHR; FRN 175042) and the Natural Sciences and Engineering Research Council of Canada (NSERC; RGPIN‐2019‐06104), awarded to BRC. HMOR is funded by a CIHR PDF award. MT is a NSERC CGSR‐M recipient.

## Conflicts of Interest

The authors declare no conflicts of interest.

## Supporting information


**Table S1:** Inclusion/exclusion criteria.

**Table S2:** Search term blocks.

**Table S3:** Bias scoring.

**Table S4:** Excluded papers.

## Data Availability

The data that support the findings of this study are available from the corresponding author upon reasonable request.
