# Supplementary material for: The Role of Hippocampal Interneuron Migration in Neurodevelopmental Disorders: A Systematic Review
Source: Hippocampus. 2026 Aug 17;36(5):e70121. doi: 10.1002/hipo.70121 (PMC13481289; doi:10.1002/hipo.70121)
Supplement: Supplementary file 1 [file HIPO-36-0-s001.docx]

**Supplementary Table 3: Bias Scoring**

|  | 1.1 | 1.2 | 1.3 | 1.4 | 1.5 | 1.6 | 1.7 | 1.8 | 1.9 | 1.10 | 1.11 | 1.12 | 1.13 | 2.0 | 2.1 | 2.2 |
| --- | --- | --- | --- | --- | --- | --- | --- | --- | --- | --- | --- | --- | --- | --- | --- | --- |
| Paper | The study addresses an appropriate and clearly focused question. | The groups being studied are selected from source populations that are comparable in all respects other than the factor under investigation. | The study indicates the sample size of each of the groups being studied. | The study reports the number of animal deaths as a result of the exposure model. | If animals died as a result of the exposure model, all efforts were made to include data from the animal in the study. | The outcomes are clearly defined. | The assessment of outcome is made blind to exposure status. If the study is retrospective this may not be applicable. | Where blinding was not possible, there is some recognition that knowledge of exposure status could have influenced the assessment of outcome. | The method of assessment of exposure is reliable. | Evidence from other sources is used to demonstrate that the method of outcome assessment is valid and reliable. | The main potential confounders are identified and taken into account in the design and analysis. | Have values for the upper and lower limits of the data been provided? | How well was the study done to minimise the risk of bias or confounding? | Taking into account your evaluation of the methodology used, and the statistical power of the study, do you think there is clear evidence of an association between exposure and outcome? | Does the study report the sex of the animals used? | Does the study consider sex as a biological variable in their study design? |
| Lussier et al., 2016 | Acceptable | Acceptable | Unacceptable | Unacceptable | Cannot say | Acceptable | Unacceptable | Unacceptable | Acceptable | Acceptable | Acceptable | Acceptable | Unacceptable | Unacceptable | Acceptable | Unacceptable |
| Dinday et al., 2017 | Acceptable | Acceptable | Acceptable | Unacceptable | Cannot say | Acceptable | Unacceptable | Unacceptable | Acceptable | Acceptable | Acceptable | Acceptable | Unacceptable | Acceptable | Acceptable | Acceptable |
| Berghuis et al., 2005 | Acceptable | Acceptable | Acceptable | Unacceptable | Cannot say | Acceptable | Unacceptable | Unacceptable | Acceptable | Acceptable | Unacceptable | Unacceptable | Unacceptable | Acceptable | Unacceptable | Unacceptable |
| Huo et al., 2018 | Acceptable | Acceptable | Acceptable | Unacceptable | Cannot say | Acceptable | Unacceptable | Unacceptable | Acceptable | Acceptable | Acceptable | Acceptable | Unacceptable | Acceptable | Unacceptable | Unacceptable |
| Liu et al., 2019 | Acceptable | Acceptable | Acceptable | Unacceptable | Cannot say | Acceptable | Unacceptable | Unacceptable | Acceptable | Acceptable | Unacceptable | Unacceptable | Unacceptable | Acceptable | Unacceptable | Unacceptable |
| Uchida et al., 2014 | Acceptable | Acceptable | Acceptable | Unacceptable | Cannot say | Acceptable | Acceptable | Acceptable | Acceptable | Acceptable | Unacceptable | Acceptable | Unacceptable | Acceptable | Unacceptable | Unacceptable |
| Eckert et al., 2021 | Acceptable | Acceptable | Acceptable | Unacceptable | Cannot say | Acceptable | Unacceptable | Unacceptable | Acceptable | Acceptable | Acceptable | Unacceptable | Unacceptable | Acceptable | Acceptable | Unacceptable |
| Santhakumar et al., 2024 | Acceptable | Acceptable | Acceptable | Unacceptable | Unacceptable | Acceptable | Acceptable | Not applicable | Acceptable | Acceptable | Acceptable | Unacceptable | Unacceptable | Acceptable | Acceptable | Unacceptable |
| Iida et al., 2024 | Acceptable | Acceptable | Acceptable | Acceptable | Cannot say | Acceptable | Unacceptable | Unacceptable | Acceptable | Acceptable | Acceptable | Unacceptable | Unacceptable | Acceptable | Unacceptable | Unacceptable |
| Pennucci et al., 2016 | Acceptable | Acceptable | Acceptable | Unacceptable | Cannot say | Acceptable | Unacceptable | Unacceptable | Acceptable | Acceptable | Acceptable | Unacceptable | Unacceptable | Acceptable | Unacceptable | Unacceptable |
| Saegusa et al., 2012 | Acceptable | Acceptable | Acceptable | Unacceptable | Cannot say | Acceptable | Unacceptable | Unacceptable | Acceptable | Acceptable | Acceptable | Unacceptable | Unacceptable | Acceptable | Acceptable | Unacceptable |
| Lee et al., 2013 | Acceptable | Acceptable | Acceptable | Unacceptable | Cannot say | Acceptable | Unacceptable | Unacceptable | Acceptable | Acceptable | Acceptable | Acceptable | Unacceptable | Acceptable | Unacceptable | Unacceptable |
| Fleck et al., 2000 | Acceptable | Acceptable | Unacceptable | Unacceptable | Cannot say | Acceptable | Unacceptable | Unacceptable | Acceptable | Acceptable | Acceptable | Unacceptable | Unacceptable | Unacceptable | Unacceptable | Unacceptable |
| Price et al., 2009 | Acceptable | Acceptable | Acceptable | Unacceptable | Cannot say | Acceptable | Unacceptable | Unacceptable | Acceptable | Acceptable | Acceptable | Acceptable | Unacceptable | Acceptable | Acceptable | Unacceptable |
| Seah et al., 2008 | Acceptable | Acceptable | Acceptable | Unacceptable | Cannot say | Acceptable | Unacceptable | Unacceptable | Acceptable | Acceptable | Unacceptable | Acceptable | Unacceptable | Acceptable | Unacceptable | Unacceptable |
| Akane et al., 2013 | Acceptable | Acceptable | Acceptable | Unacceptable | Cannot say | Acceptable | Unacceptable | Unacceptable | Acceptable | Acceptable | Acceptable | Unacceptable | Unacceptable | Acceptable | Acceptable | Unacceptable |
| Fulp et al., 2008 | Acceptable | Acceptable | Acceptable | Unacceptable | Cannot say | Acceptable | Not applicable | Not applicable | Acceptable | Acceptable | Acceptable | Unacceptable | Acceptable | Acceptable | Unacceptable | Unacceptable |
| Kim et al., 2023 | Acceptable | Acceptable | Acceptable | Unacceptable | Cannot say | Acceptable | Acceptable | Not applicable | Acceptable | Acceptable | Unacceptable | Acceptable | Unacceptable | Acceptable | Acceptable | Unacceptable |
| Giovanoli et al., 2014 | Acceptable | Acceptable | Acceptable | Unacceptable | Cannot say | Acceptable | Acceptable | Not applicable | Acceptable | Acceptable | Acceptable | Unacceptable | Acceptable | Acceptable | Acceptable | Unacceptable |
| Stevens et al., 2013 | Acceptable | Acceptable | Acceptable | Unacceptable | Cannot say | Acceptable | Acceptable | Not applicable | Acceptable | Acceptable | Acceptable | Unacceptable | Acceptable | Acceptable | Acceptable | Unacceptable |
| Berden et al., 2025 | Acceptable | Acceptable | Acceptable | Unacceptable | Cannot say | Acceptable | Unacceptable | Unacceptable | Acceptable | Acceptable | Acceptable | Unacceptable | Unacceptable | Acceptable | Unacceptable | Unacceptable |
| Nakai et al., 2014 | Acceptable | Acceptable | Acceptable | Unacceptable | Cannot say | Acceptable | Acceptable | Not applicable | Acceptable | Acceptable | Acceptable | Unacceptable | Acceptable | Acceptable | Acceptable | Acceptable |
| Cobos et al., 2005 | Acceptable | Acceptable | Acceptable | Acceptable | Unacceptable | Acceptable | Unacceptable | Unacceptable | Acceptable | Acceptable | Acceptable | Acceptable | Unacceptable | Acceptable | Unacceptable | Unacceptable |
| McManus et al., 2004 | Acceptable | Acceptable | Acceptable | Unacceptable | Unacceptable | Acceptable | Unacceptable | Unacceptable | Acceptable | Acceptable | Acceptable | Unacceptable | Unacceptable | Acceptable | Unacceptable | Unacceptable |
| Lawrence et al., 2010 | Acceptable | Acceptable | Acceptable | Not applicable | Not applicable | Acceptable | Unacceptable | Unacceptable | Acceptable | Acceptable | Acceptable | Unacceptable | Acceptable | Acceptable | Acceptable | Unacceptable |
| Zhu et al., 2021 | Acceptable | Acceptable | Acceptable | Unacceptable | Unacceptable | Acceptable | Acceptable | Not applicable | Acceptable | Acceptable | Acceptable | Unacceptable | Acceptable | Acceptable | Unacceptable | Unacceptable |
| Steinecke et al., 2012 | Acceptable | Acceptable | Acceptable | Unacceptable | Unacceptable | Acceptable | Unacceptable | Unacceptable | Acceptable | Acceptable | Acceptable | Acceptable | Unacceptable | Unacceptable | Unacceptable | Unacceptable |
| Colombo et al., 2007 | Acceptable | Acceptable | Acceptable | Unacceptable | Unacceptable | Acceptable | Acceptable | Not applicable | Acceptable | Acceptable | Acceptable | Acceptable | Acceptable | Acceptable | Unacceptable | Unacceptable |
| Shen et al., 2025 | Acceptable | Acceptable | Acceptable | Unacceptable | Unacceptable | Acceptable | Unacceptable | Unacceptable | Acceptable | Acceptable | Acceptable | Unacceptable | Unacceptable | Acceptable | Acceptable | Unacceptable |
