# Supplementary material for: The Role of Hippocampal Interneuron Migration in Neurodevelopmental Disorders: A Systematic Review
Source: Hippocampus. 2026 Aug 17;36(5):e70121. doi: 10.1002/hipo.70121 (PMC13481289; doi:10.1002/hipo.70121)
Supplement: Supplementary file 2 [file HIPO-36-0-s002.docx]

**Supplementary Table 2: Search Term Blocks**

| Reason for Terms | Terms Used |
| --- | --- |
| Specifies interneuron: | “interneuron” OR “gabaergic” OR “GABA” OR “parvalbumin” OR “NP-Y” OR “somatostatin” or “GABAA” OR “GABAB” OR “neuropeptide-y” OR “neuropeptide Y” OR “calbindin” OR “CCK” OR “cholecystokinin” OR “calretinin” OR “GAD67” OR “GAD65” OR “Glutamate decarboxylase”OR “nNOS” OR “reelin” OR “5HTR3a” |
| Specifies interneuron migration: | “Migration” OR “migrating” OR “migrated” OR “migrate” OR “CGE” OR “caudal ganglionic eminence” OR “MGE” OR “medial ganglionic eminence” OR “morphogenesis” |
| Specifies hippocampus: | “hippocampus” OR “hippocampi” OR “limbic system” OR “limbic circuit” OR “long term plasticity” or “long term depression” OR “circuit of Papez” OR “cornu ammonis” OR “CA” OR “dentate gyrus” OR “dentate” OR “DG” OR “entorhinal cortex” OR “EC” OR “perforant pathway” OR “fimbria-fornix” OR “Schaffer collaterals” OR “commissural pathway” |
