# Supplementary material for: The Role of Hippocampal Interneuron Migration in Neurodevelopmental Disorders: A Systematic Review
Source: Hippocampus. 2026 Aug 17;36(5):e70121. doi: 10.1002/hipo.70121 (PMC13481289; doi:10.1002/hipo.70121)
Supplement: Supplementary file 3 [file HIPO-36-0-s003.docx]

**Supplementary Table 4: Excluded Papers**

| Paper Name | First Author | Year | Reason |
| --- | --- | --- | --- |
| Reelin regulates the migration of late-born hippocampal CA1 neurons via cofilin phosphorylation. | Ishii | 2023 | Not about interneuron migration |
| Sialyltransferase Mutations Alter the Expression of Calcium-Binding Interneurons in Mice Neocortex, Hippocampus and Striatum. | Blažetić | 2023 | Not about interneuron migration |
| Neonatal isoflurane exposure disturbs granule cell migration in the rat dentate gyrus. | Uchida | 2022 | Not about neurodevelopmental disorders |
| Interneuron Heterotopia in the Lis1 Mutant Mouse Cortex Underlies a Structural and Functional Schizophrenia-Like Phenotype. | Garcia-Lopez | 2021 | Not about interneuron migration |
| Epilepsy-induced motility of differentiated neurons. | Chai | 2014 | Not about interneuron migration |
| Reelin regulates neuronal progenitor migration in intact and epileptic hippocampus. | Gong | 2007 | Not about interneuron migration |
| Developmental role of the cell adhesion molecule Contactin-6 in the cerebral cortex and hippocampus. | Zuko | 2016 | Not about interneuron migration |
| Influence of nitrous oxide on granule cell migration in the dentate gyrus of the neonatal rat. | Saito | 2018 | Not about interneuron migration |
| Ephrin Bs are essential components of the Reelin pathway to regulate neuronal migration. | Sentürk | 2011 | Not about interneuron migration |
| Intersectin 1 is a component of the Reelin pathway to regulate neuronal migration and synaptic plasticity in the hippocampus. | Jakob | 2017 | Not about interneuron migration |
| Sustained production of Reelin-expressing interneurons in the hippocampal dentate hilus after developmental exposure to anti-thyroid agents in rats. | Saegusa | 2010 | Not about interneuron migration |
| Alpha-CaMKII deficiency causes immature dentate gyrus, a novel candidate endophenotype of psychiatric disorders. | Yamasaki | 2008 | Not about interneuron migration |
| Accumulation of GABAergic neurons, causing a focal ambient GABA gradient, and downregulation of KCC2 are induced during microgyrus formation in a mouse model of polymicrogyria. | Wang | 2014 | Not in the hippocampus |
| Decreased reelin expression in the left prefrontal cortex (BA9) in chronic schizophrenia patients. | Habl | 2012 | Not about interneuron migration |
| Reversible aberration of neurogenesis targeting late-stage progenitor cells in the hippocampal dentate gyrus of rat offspring after maternal exposure to acrylamide. | Ogawa | 2012 | Not about interneuron migration |
| No effect of sustained systemic growth retardation on the distribution of Reelin-expressing interneurons in the neuron-producing hippocampal dentate gyrus in rats. | Ohishi | 2010 | Not about interneuron migration |
| Physiological and morphological characterization of dentate granule cells in the p35 knock-out mouse hippocampus: evidence for an epileptic circuit. | Patel | 2004 | Not about interneuron migration |
| A Peptide Link Between Human Cytomegalovirus Infection, Neuronal Migration, and Psychosis. | Lucchese | 2020 | Not about interneuron migration |
| Autosomal recessive lissencephaly with cerebellar hypoplasia is associated with human RELN mutations. | Hong | 2000 | Not about interneuron migration |
| Transient downregulation of Dab1 protein levels during development leads to behavioral and structural deficits: relevance for psychiatric disorders. | Teixeira | 2014 | Not about interneuron migration |
| Role for reelin in the development of granule cell dispersion in temporal lobe epilepsy. | Haas | 2002 | Not about interneuron migration |
| Delayed cell migration in the developing rat brain following maternal omega 3 alpha linolenic acid dietary deficiency. | Yavin | 2009 | Not about interneuron migration |
| Maternal single injection of N-methyl-N-nitrosourea to cause microcephaly in offspring induces transient aberration of hippocampal neurogenesis in mice. | Takimoto | 2014 | Not about interneuron migration |
| Underexpression of neural cell adhesion molecule and neurotrophic factors in rat brain following thromboxane A(2)-induced intrauterine growth retardation. | Fukami | 2000 | Not about interneuron migration |
| Mutations of CNTNAP1 led to defects in neuronal development. | Li | 2020 | Not about interneuron migration |
| Effects of miR-137 on pyramidal neuron morphology and GABA interneuron neurogenesis | Xu | 2023 | Not peer reviewed |
| The Role of Redox Dysregulation in the Effects of Prenatal Stress on Embryonic Interneuron Migration | Bittle | 2019 | Not in the hippocampus |
| Sex Differences in Affective Dysfunction and Alterations in Parvalbumin in Rodent Models of Early Life Adversity | Ellis | 2021 | Review paper |
| Parvalbumin and parvalbumin chandelier interneurons in autism and other psychiatric disorders | Juarez | 2022 | Review paper |
| Graded reduction of Pafah1b1 (Lis1) activity results in neuronal migration defects and early embryonic lethality | Hirotsune | 1998 | Not about interneuron migration |
| A selective decrease in the relative density of parvalbumin-immunoreactive neurons in the hippocampus in schizophrenia | Zhang | 2002 | Not about interneuron migration |
| Glutamate Decarboxylase 67 Deficiency in a Subset of GABAergic Neurons Induces Schizophrenia-Related Phenotypes | Fujihara | 2015 | Not about interneuron migration |
| Stem cell-derived interneuron transplants as a treatment for schizophrenia: Preclinical validation in a rodent model | Donegan | 2017 | Not about interneuron migration |
| Hippocampal interneuron transplants reverse aberrant dopamine system function and behavior in a rodent model of schizophrenia | Perez | 2013 | Not about interneuron migration |
| Knockdown of Lhx6 during embryonic development results in neurophysiological alterations and behavioral deficits analogous to schizophrenia in adult rats. | Elam | 2024 | Not about interneuron migration |
| Postnatal development and migration of cholecystokinin-immunoreactive interneurons in rat hippocampus. | Morozov | 2003 | Not about neurodevelopmental disorders |
