# Supplementary material for: The Role of Hippocampal Interneuron Migration in Neurodevelopmental Disorders: A Systematic Review
Source: Hippocampus. 2026 Aug 17;36(5):e70121. doi: 10.1002/hipo.70121 (PMC13481289; doi:10.1002/hipo.70121)
Supplement: Supplementary file 4 [file HIPO-36-0-s004.docx]

**Supplementary Table 1: Inclusion/ Exclusion Criteria**

| Inclusion | Exclusion |
| --- | --- |
| - Must be in English - Must be a peer-reviewed journal article - Must not be a review - Must not be a case study - Must not be a conference poster or abstract submission - Must be in the context of neurodevelopmental disorders - Must include results specific to the hippocampus - Must be specific to interneuron migration | - Not in English - Not a peer-reviewed journal article - Is a review - Is a case study - Is a conference poster or abstract submission - Is not in the context of neurodevelopmental disorders - Does not include results specific to the hippocampus - Does not include results specific to interneuron migration |
